# The tumor immune microenvironment of primary and metastatic HER2− positive breast cancers utilizing gene expression and spatial proteomic profiling

**DOI:** 10.1186/s12967-021-03113-9

**Published:** 2021-11-27

**Authors:** Ilana Schlam, Sarah E. Church, Tyler D. Hether, Krysta Chaldekas, Briana M. Hudson, Andrew M. White, Emily Maisonet, Brent T. Harris, Sandra M. Swain

**Affiliations:** 1 Department of Hematology-Oncology, MedStar Washington Hospital Center, Washington, DC USA; 2 Present Address: Department of Hematology and Oncology, Tufts Medical Center, 800 Washington St, 245, Boston, MA 02111 USA; 3 NanoString Technologies Inc., Seattle, WA USA; 4 MedStar Georgetown University Hospital, 4000 Reservoir road NW, 120 Building D, Washington, DC 20057 USA; 5 Lombardi Comprehensive Cancer Center, Washington, DC USA; 6 MedStar Health, Washington, DC USA

**Keywords:** Breast cancer, HER2 positive, Tumor microenvironment, Digital spatial profiling, Gene expression profiling

## Abstract

**Background:**

The characterization of the immune component of the tumor microenvironment (TME) of human epidermal growth factor receptor 2 positive (HER2+) breast cancer has been limited. Molecular and spatial characterization of HER2+ TME of primary, recurrent, and metastatic breast tumors has the potential to identify immune mediated mechanisms and biomarker targets that could be used to guide selection of therapies.

**Methods:**

We examined 15 specimens from eight patients with HER2+ breast cancer: 10 primary breast tumors (PBT), two soft tissue, one lung, and two brain metastases (BM). Using molecular profiling by bulk gene expression TME signatures, including the Tumor Inflammation Signature (TIS) and PAM50 subtyping, as well as spatial characterization of immune hot, warm, and cold regions in the stroma and tumor epithelium using 64 protein targets on the GeoMx Digital Spatial Profiler.

**Results:**

PBT had higher infiltration of immune cells relative to metastatic sites and higher protein and gene expression of immune activation markers when compared to metastatic sites. TIS scores were lower in metastases, particularly in BM. BM also had less immune infiltration overall, but in the stromal compartment with the highest density of immune infiltration had similar levels of T cells that were less activated than PBT stromal regions suggesting immune exclusion in the tumor epithelium.

**Conclusions:**

Our findings show stromal and tumor localized immune cells in the TME are more active in primary versus metastatic disease. This suggests patients with early HER2+ breast cancer could have more benefit from immune-targeting therapies than patients with advanced disease.

**Supplementary Information:**

The online version contains supplementary material available at 10.1186/s12967-021-03113-9.

## Background

Human epidermal growth factor receptor 2 positive (HER2+) breast cancer accounts for 20–25% of all breast cancers [1, 2]. These tumors were historically associated with poor prognosis [1, 2]. With more effective HER2 targeted therapy the outcomes of these patients have improved markedly [3, 4]. However, there is still an unmet need to prevent recurrences in patients with early stage breast cancer [5, 6] and to achieve more durable responses in those with metastatic disease [7–9].

The molecular subtypes and spatial interactions of immune, stroma, and tumor cells in the tumor microenvironment (TME) are not well defined in HER2+ breast cancer. HER2 is a natural antigen and the effect of some HER2-directed therapies could be potentiated with immune checkpoint inhibitors (ICI) [10, 11]. A better understanding of molecular biomarkers and spatial characterization of immune cells in HER2+ breast cancer environment could help for selection of combination therapies, targeting both HER2 and the immune components, that would be most effective [10, 11]. Metastatic triple negative breast cancers (TNBC) have been shown to be more immunologically quiescent than primary tumors though deep profiling of spatial characterization of immune cells in HER2+ disease has yet to be done [12–14].

Immunotherapy has revolutionized the treatment paradigm of several malignancies but its use has been limited in breast cancer and mostly studied in the TNBC subtype [15–17]. Two ICIs have been approved in combination with chemotherapy for selected patients with advanced TNBC; program cell death ligand 1 (PD-L1) expression allows for the selection of patients that more likely to respond to ICI [15, 16]. The combination of HER2 targeted agents with ICI have shown promising results in patients with advanced PDL1-positive disease and there is growing interest in utilizing these treatments for this breast cancer subtype [18, 19]. Since PD-L1 assays identify a single immune checkpoint pathway, further characterization of the state of immune cells in the TME at different stages of disease both by bulk gene expression profiling and spatial analysis could allow us to identify mechanisms of tumor immune escape and other potential therapeutic targets [20].This could help determine the optimal combinations as well as the stage and line of treatment in which immunotherapy is most effective.

In this study we utilized bulk tissue gene expression profiling [21] and digital spatial profiling (DSP) of proteins to characterized the TME of HER2+ primary and metastatic tumors from multiple patients and tissue regions to understand changes in immune contexture as tumors evolve after metastatic migration during therapy.

## Materials and methods

### Patients and samples

Samples of patients with HER2+ breast cancer between 2001 and 2019 were analyzed by the Georgetown University Histopathology Tissue Shared Resource and 15 formalin fixed paraffin embedded (FFPE) samples were selected for this study. Deidentified archival samples were selected through Georgetown University Hospital Institutional Review Board (IRB) approved protocols (1992-048, 2007-345 and Pro00000007).

Samples included 15 specimens from eight patients. Histopathology of the specimens was obtained from the original pathology reports as were results of estrogen receptor (ER), progesterone receptor (PR), and HER2 status.

### RNA extraction and gene expression profiling

RNA was extracted from one slide of unstained FFPE tissue and macro-dissection to remove non-tumor was performed based on a sequential hematoxylin and eosin (H&E) staining. Approximately 50-200 ng of RNA was run on NanoString’s nCounter PanCancer IO 360™ assay (IO360) containing 770 genes with an additional 28-gene PAM50 spike-in. Genes were normalized using a combination of geometric means of housekeeping (HK) gene expression and an IO360 panel standard run on the same cartridge. IO360 signatures were calculated as previously described [22, 23].Calculation of the PAM50 subtypes and Tumor Inflammation Signature (TIS) scores were done as previously described [22, 23]. TIS status was binned into high and low categories based on the geometric mean of the cohort (low < TIS score 6.5 < high). Genes were normalized using a ratio of the expression value to the geometric mean of all HK genes on the panel.

### GeoMx digital spatial profiling

FFPE tissue from eight patients were profiled using GeoMx DSP as previously described [24]. An H&E slide was used to guide identification of tumor regions to select regions of interest (ROIs). Immunofluorescent visualization marker for Pan-Cytokeratin (PanCK, tumor), CD45 (immune) and CD3 (T cells) were used to guide selection of hot (highly infiltrated tumor regions), warm (partially infiltrated tumor regions) and cold (regions with low infiltration) ROIs. Each ROI was segmented for tumor (PanCK +) or stroma (PanCK-) areas of illumination (AOIs). For each AOI, 70 protein targets including housekeeping proteins and isotype controls from the following cores and modules [(v1.0) Human Immune Cell Profiling Protein Core, (v0.9) Human Cell Death Protein, (v1.0) Human Immune Activation Status Protein, (v1.0) Human IO Drug Target Protein, (v1.0) Human Pan-Tumor Protein, (v0.9) Human PI3K/AKT Signaling Protein, NanoString Technologies] were measured Additional file 1: Table S1. The signal-to-noise ratio for each of the targets included is shown in Additional file 1: Figure S1. Protein data were scaled to area and normalized using two housekeeping proteins (S6, Histone H3) using GeoMx DSP Analysis version 2.2.0.64.

### Statistical analysis

#### Gene expression

Differential gene expression (nCounter) is fit on a per gene or per signature basis using a linear mixed model for analyses with subject as a blocking factor to account for the temporal effects in the model. The statistical model uses the expression value or signature score as the dependent variable and fits a grouping variable as a fixed effect to test for differences in the levels of that grouping variable. The duplicate Correlation function within the limma [25] R package is used to assess the correlation between subsequent time points. This correlation estimate is fit into the linear mixed effect model with subject as the random effect and the correlation between the repeated temporal measurements.

P-values are adjusted within each analysis, gene or signature, and on the grouping variable level difference t-test using the Benjamini and Yekutieli False Discovery Rate (FDR) adjustment to account for correlations amongst the tests (FDR of 5%). All gene expression models are fit using the limma package in R [25].

#### GeoMx digital spatial profiling

For the GeoMx DSP protein data, hierarchical clustering analyses were performed to look for broad patterns within and between subjects based on TIS status, PAM50 subtype, timepoint (primary or metastatic), ROI type (immune cold, warm, hot), and AOI type (tumor, PanCK + ; stroma, PanCK-). For hierarchical clustering, the HK protein-based normalized expression values were first log2 transformed and the resulting values were then centered and scaled. The Z-scores for each protein were then used as input to the hierarchical clustering in the R package pheatmap [26].

Differential expression analyses for DSP data were used to look for specific protein differences between three different groupings: (1) Primary vs metastatic tumor, (2) Breast vs brain tumors, and (3) timepoint (three different samples of a single patient). To account for multiple observations (i.e., ROIs) within a given patient, a linear mixed effects model was used in the R package lmerTest [27]. Specifically, the log2 transformed normalized expression values for a given protein were used as the dependent variable and a single fixed effect was used (e.g., primary vs metastatic; 2 levels). Patient ID was used as the random effect (with random intercept). For each resulting model (1 model for each of the 64 proteins -plus housekeeping proteins and isotype controls-), the degrees of freedom were estimated using Satterthwaite's method [27].To account for multiple hypothesis testing (*i.e*., 64 hypotheses), raw P-values were adjusted based on an FDR level of 0.05 using the Benjamini-Hochberg (BH) procedure [28]. This procedure was done for the primary vs metastatic tissue comparison and for the narrower breast vs brain comparisons—both of which had two levels. To compare time series data, a single model for each protein was used with timepoint as a single fixed effect with three levels: Primary 1, Primary 2, and Metastatic. As before, Patient ID was used as a random effect to account for multiple observations within a subject. For each model, the three pairwise log2 fold changes were derived using the marginal means method (*i.e.,* “least squared means”) in lmerTest. BH P-value adjustment for time series data was performed for a given pairwise contrast (e.g., Primary 1 vs Primary 2).

Boxplots were used to better visualize the protein expression for a given patient across time using the R package ggplot2 [29]. This was done for 2-time point and 3-time point data (Table 1).

**Table 1:** Summary of patient and tumor characteristics

Patient	Tissue	Diagnosis	ER(%)/PR(%)/HER2( +)	PAM50 Subtype	TIS Status	Treatment at the time of sample collection
1	Breast	IDC, DCIS	2/0/3	Luminal A	High	Gemcitabine and trastuzumab
1	Breast	Tumor emboli	N/A	Luminal A	High	Nab-paclitaxel and trastuzumab
1	Lung	Metastatic	0/0/3	HER2 enriched	High	Nab-paclitaxel and trastuzumab
2	Breast	ICD, DCIS	0/0/3	HER2 enriched	Low	None. Initial diagnosis
2	Breast	Residual IDC	0/0/N/A	HER2 enriched	High	TDM1, pertuzumab, doxorubicin and cyclophosphamide*
2	Soft tissue	Local recurrence	0/0/3	HER2 enriched	Low	Trastuzumab and pertuzumab
3	Brain	Metastatic	15/0/3	HER2 enriched	Low	None. Discontinued due to toxicities
4	Breast	IDC	0/0/3	Basal like	High	None. Initial diagnosis
4	Breast	Local recurrence	N/A	Luminal A	High	Doxorubicin, cyclophosphamide, paclitaxel and trastuzumab
5	Brain	Metastatic	0/0/3	Basal like	Low	TDM1 and anastrozole
6	Breast	IDC, DCIS	80/70/2**	Luminal A	High	None. Initial diagnosis
7	Breast	IDC, DCIS	0/0/3	HER2 enriched	High	None. Initial diagnosis
7	Breast	Local recurrence	N/A	Basal like	High	None. Completed neoadjuvant and adjuvant therapy
8	Breast	IDC, DCIS	10/15/1	Luminal A	Low	None. Initial diagnosis
8	Soft tissue	Local recurrence	0/0/3	Basal like	Low	Nab-paclitaxel and atezolizumab (previously had triple negative disease)

DCIS: ductal carcinoma in situ; DCIS: ductal carcinoma in situ; ER: estrogen receptor; HER2: human epidermal growth factor 2; IDC: invasive ductal carcinoma; IDC: invasive ductal carcinoma; PR: progesterone receptor; TDM1: ado-trastuzumab emtansine; TIS: tumor inflammation score

^*^Neoadjuvant therapy in the ISPY2 clinical trial

^**^HER2/CEP17 ratio: 2.13 (amplified by FISH)

## Results

### Study cohort

Fifteen specimens from eight patients were analyzed, including 10 primary breast tumors, two soft tissue tumors, two brain metastases and one lung metastasis. Eight patients were receiving cancer-directed therapies (details in Table 1 and Additional file 1: Figures S2 and S3) at the time of the sample collection while seven were not. Two of the patients had three matching timepoints, three patients had two matching timepoints. Finally, three patients had a single time point each which included two patients with brain metastases. Additional patient and sample characteristics are summarized on Table 1.

### Primary versus metastatic tumors

**Fig. 1:**
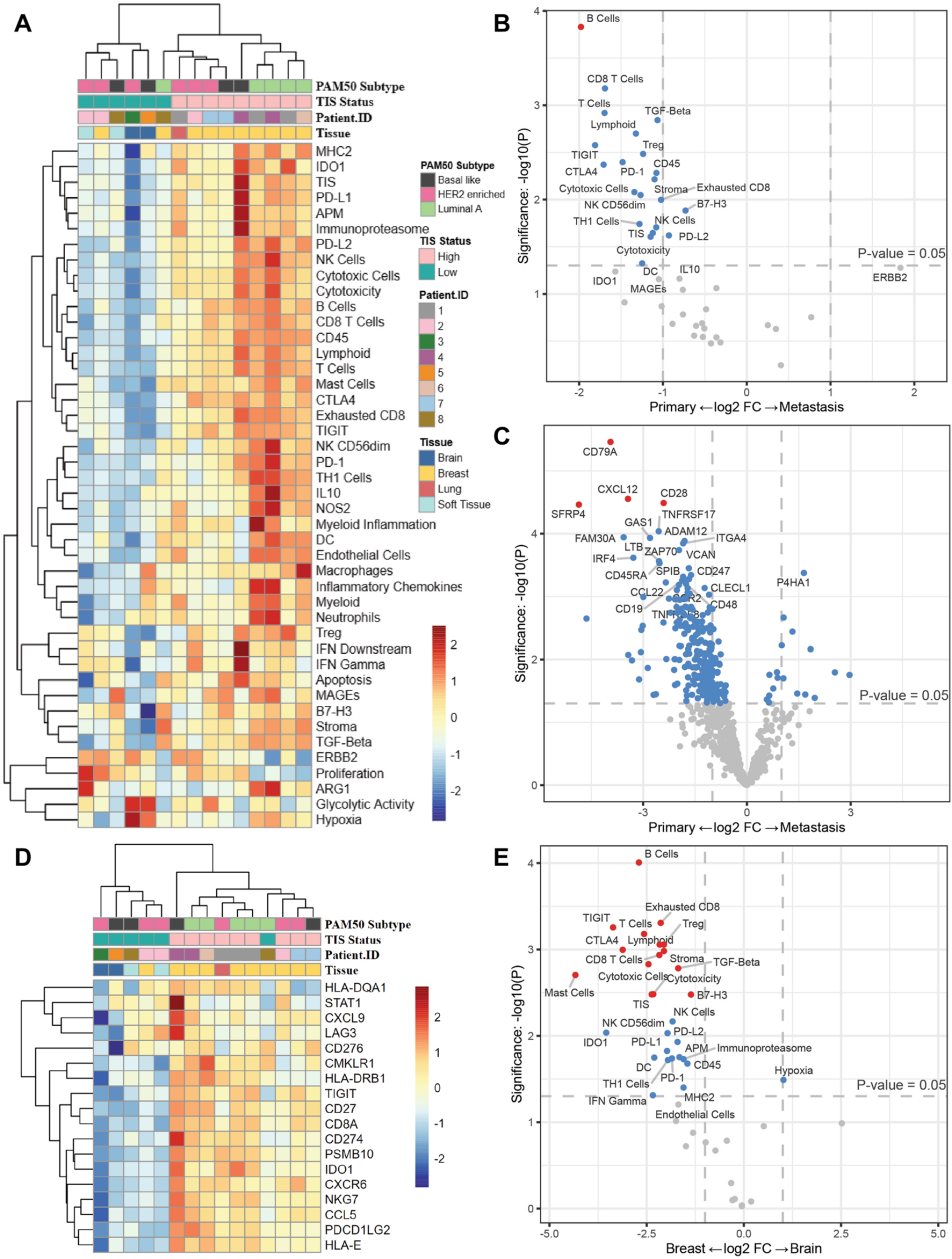
IO360 gene expression analysis of primary and metastatic lesions. **A** Unsupervised hierarchical clustering of IO360 signatures for each sample. **B** Differential expression of IO360 signatures comparing primary breast and metastatic samples. **C** Differential expression of IO360 genes comparing primary breast and metastatic samples. **D** Unsupervised hierarchical clustering of the 18 TIS genes. **E** Differential expression of IO360 genes comparing primary breast and brain metastases. For B,C,E vertical dotted lines represent a onefold log change and the horizontal line marks an unadjusted p-value of p < 0.05. Dots in grey are not significant, dots in blue have an unadjusted p-value of < 0.05 and dots in red have an unadjusted p-value of < 0.05

We compared all primary breast cancer tumors to each metastatic tissue to look at differential expression of genes and protein by sample and tissue type. We examined gene expression, IO360 signatures, TIS status (which measures a periphery suppressed anti-tumor immune response) and PAM50 subtypes from bulk tumor tissue (Fig. 1a).

Metastatic disease and primary samples clustered separately based on IO360 signatures, with 8/10 primary tumors having high TIS score status (Fig. 1a, Table 1). The B cell abundance signature was significantly higher in the primary compared to metastatic tumors (Fig. 1B). Several lymphocyte-related signatures trended toward having higher expression in primary tumors including TIGIT, CTLA4, T cells, CD8 T cells, PD1, cytotoxic cells, lymphoid cells, TH1, natural killer cells, cytotoxicity, TIS, exhausted CD8, PD-L2, and B7-H3, but did not reach statistical significance with an adjusted p-value (Fig. 1B, Additional file 1: Table S2). Individual genes associated with immune activation and trafficking were significantly higher (adjusted p-value < 0.05) in primary tumors, including CD79A, CXCL12, CD28, and, SFRP4 (Fig. 1C, Additional file 1: Table S3). When examining the 18 individual genes in TIS, there was clustering of the metastatic tissue from brain having the lowest expression of all genes (Fig. 1D). There was also a significantly higher expression of immune signatures including B cells, exhausted CD8, TIGIT, T cells, T regulatory, lymphoid, stroma, CTLA4, CD8 T cells, cytotoxic cells, TGF-beta, mast cells, cytotoxicity, TIS, and B7H3 in primary tumors compared to brain metastases (Fig. 1E, Additional file 1: Table S4).

**Fig. 2:**
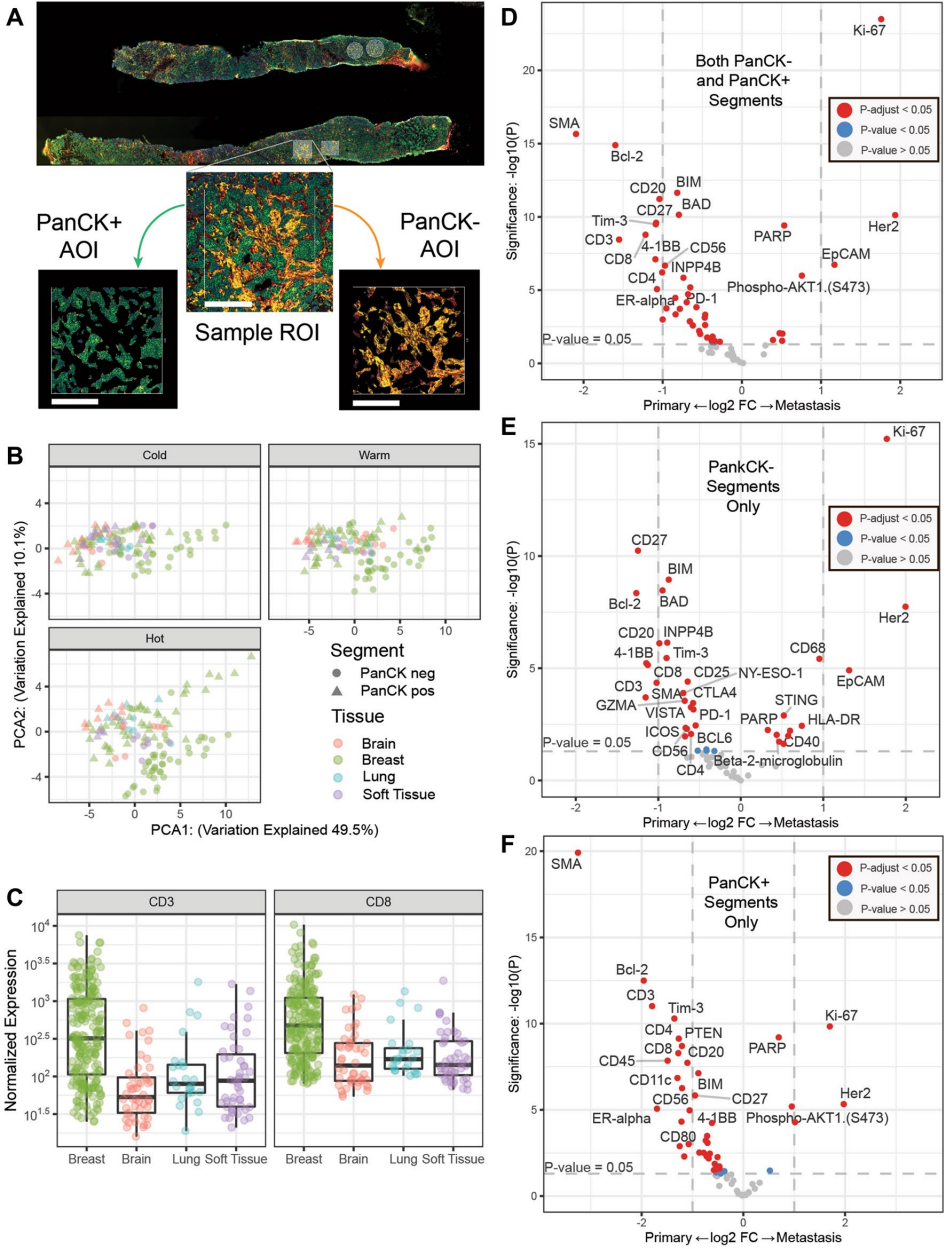
Digital spatial profiling of primary and metastatic tumors. **A** Visualization markers for Pan-Cytokeratin (PanCK, tumor—green), CD45 (total immune—red) and CD3 (T cells—yellow). Each ROI segmented tumor (PanCK +) or stroma (PanCK-) areas of illumination (AOI). Scale bar (white) is 250 microns. **B** Normalized Protein expression of CD3 and CD8 in primary and each metastatic site (lung, brain and soft tissue). **C** Principle components analysis of the 30 proteins with the highest coefficient of variation faceted by primary vs metastatic site and immune status (cold, warm and hot). **D** Differential expression of proteins from all ROI types and segments comparing primary breast and all metastatic. **E** Differential expression of proteins from all ROI types from the PanCK negative stromal segments comparing primary breast and all metastatic. **F** Differential expression of proteins from all ROI types from the PanCK positive tumor segments comparing primary breast and all metastatic. For D-F vertical dotted lines represent a onefold log2 change and the horizontal line marks an unadjusted p-value of p < 0.05. Dots in grey are not significant, dots in blue have an unadjusted p-value of < 0.05 and dots in red have an adjusted p-value of < 0.05

We then looked for differences between primary and metastatic lesions by spatially profiling ROIs of varying immune infiltrate (*i.e.*, cold, warm, hot) in tumor rich (PanCK +) and stromal (PanCK−) compartments. A schematic of the DSP experimental design is shown in Fig. 2A. When combining tumor and stroma compartments to look at immune hot, warm and cold regions by primary and metastatic status and sample tissue location, breast, lung, brain and soft tissue we saw higher expression of immune-related protein in primary tumors. Especially for tumor infiltrating lymphocytes (CD3, CD8) relative to any other metastatic tissue (Fig. 2B). Hot, warm and cold ROIs from each of the patients revealed varied immune infiltration in the cohort. Primary tumors had the highest number of immune cells and the metastatic site the lowest (Fig. 2B; Primary and metastatic ROIs collapsed via geometric mean; CD3: Wilcox test W = 30, P < 0.004; CD8: Wilcox test W = 29, P < 0.009).

Figure 2C shows principle components analysis of the 30 proteins with the highest coefficient of variation faceted by primary vs metastatic site, ROI immune status (cold, warm and hot) and AOI type (PanCK + or −). In ROIs of each immune type tumor AOIs cluster separately from metastatic samples despite tissue type. Interestingly, in immune hot ROIs primary breast samples not only cluster separately from metastatic samples they also have clear differences in protein expression in the stromal (PanCK-) and tumor (PanCK +) segments.

Both tumor and stroma regions of primary tumors had higher expression of immune activation and checkpoint markers—including Tim-3, CD27 and 4-1BB—compared to metastases located in all tissues (Fig. 2D). Metastatic tumors had higher expression of HER2 and proliferative marker Ki-67. We also found that B cell related proteins (CD20, BAD, BCL-2, CD27, BIM) were most highly expressed in the stromal compartments (Fig. 2E) while expression of lymphocyte-related markers (CD3, CD8, CD4, Tim-3) were elevated in tumor compartments (Fig. 2F, Additional file 1: Table S5).

We assessed the TIS for each sample. Nine of our samples had high TIS (> 6.5) and six had low TIS (< 6.5). One patient (patient 2) had discordant TIS, meaning that the primary tumor and soft tissue recurrence had low TIS status and the residual disease had high TIS status (Table 1). Finally, we assessed the four intrinsic PAM50 subtypes: Luminal A, Luminal B, HER2-enriched (HER2-E) or basal-like [30, 31] and identified six (40%) HER2-E, five (33%) Luminal A, and four (27%) basal like tumors in our cohort, no Luminal B tumors were identified (Table1). Four out of five patients with paired samples had discordant PAM50 subtypes between primary and metastatic tumors (Table 1). Then we analyzed gene signatures for each of the PAM50 subtypes within our cohort (Additional file 1: Figure S4) showing HER2E subtypes had higher proliferation and ERBB2 compared to Luminal A (adjusted p = 0.0004 and 0.017, respectively). The Luminal A tumors had higher expression of TH1 cells, PD1, dendritic cells, stroma, IL10 (Additional file 1: Figure S4). There was a trend towards higher expression of CD8 T cells, endothelial cells, T cells, inflammatory chemokines, natural killer CD56 dim, B-cells, NOS2, and TGF-beta signatures in Luminal A tumors. There were not statistically significant differences between HER2-E and Basal like tumors (Additional file 1: Figure S4).

### Paired samples

Three patients in our cohort had samples from two timepoints (Table 1; Additional file 1: Figure S3). Patient 4 was diagnosed with HER2+ early breast cancer (initial sample), received neoadjuvant chemotherapy and HER2-targeted therapy and then was found to have residual disease (second sample). Patient 7 was diagnosed with early stage breast cancer (initial sample), received neoadjuvant chemotherapy and HER2-targeted therapy which was followed by surgery and trastuzumab and two years after the completion of these therapy she developed a local recurrence (second sample). Patient 8 was diagnosed with a left breast HER2+ breast cancer (initial sample) and after 13 years she was diagnosed with a left breast TNBC for which she received neoadjuvant chemotherapy followed by surgery and adjuvant capecitabine. Two years later, she developed a TNBC local recurrence (second sample). There were differences in the expression of immune protein in these samples, patient 4 and 7 had a more similar history and we saw an increase in Bcl-2, CD20, LAG3, MET (PanCK-), PTEN, AKT (PanCK−) after therapy, the protein expression of patient 8 is different however this patient was heavily pretreated and the samples are over 15 years apart and the tumor was TNBC (Additional file 1: Figure S3).

Two of the patients analyzed in this study had specimens from three timepoints, biopsy from primary tumor, residual disease after neoadjuvant chemotherapy, and later advanced metastatic disease (Additional file 1: Figure S2). We performed a matched analysis to identify changes in the TME after therapy and after undergoing metastatic migration.

**Fig. 3:**
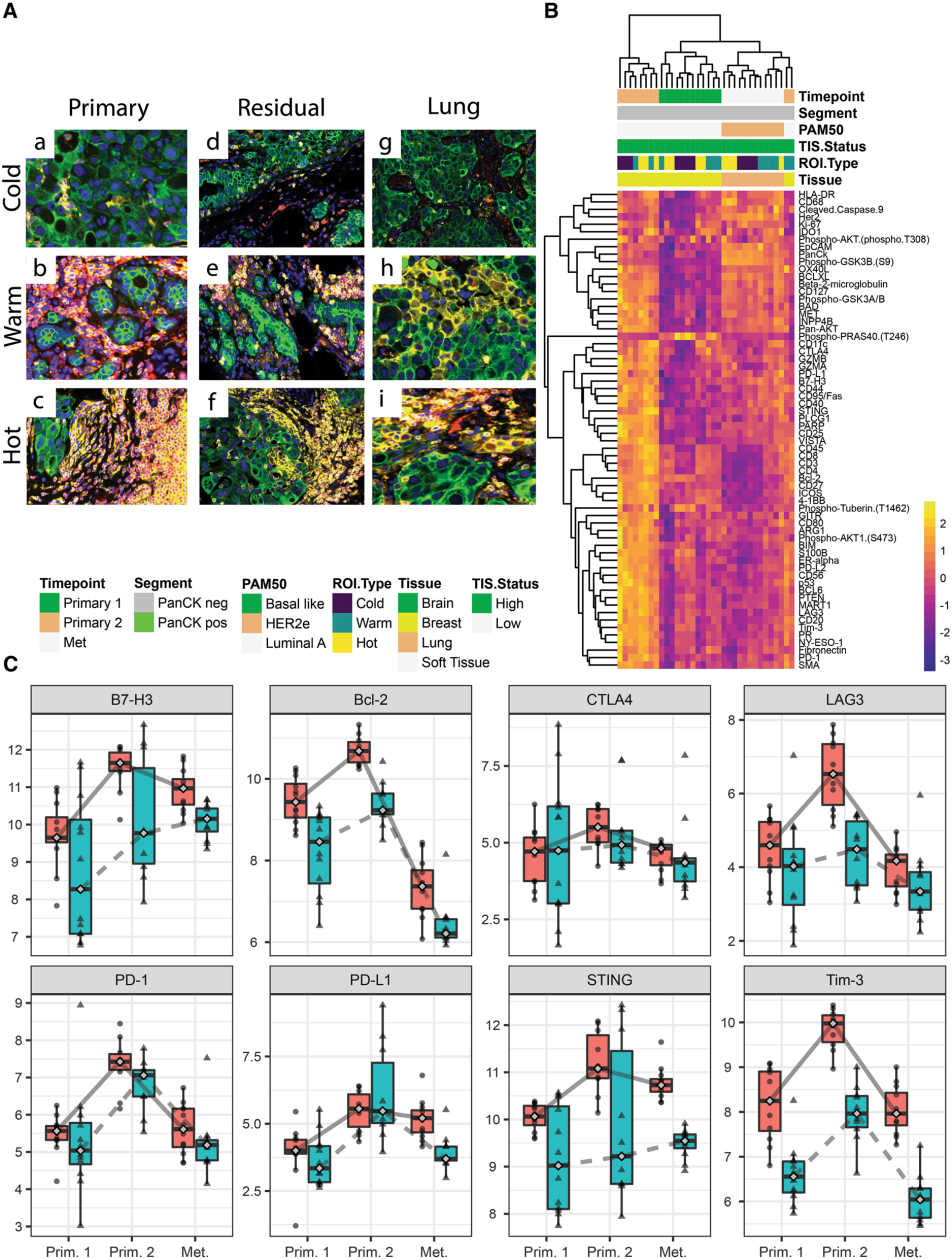
Analysis of primary, residual breast tumor and metastatic of patient 1. **A** Shows immune cold, warm and hot regions of interest for the three timepoints for patient 1 at primary, residual breast tumor and metastatic stages. Pan-cytokeratin is stained in green, CD45 in red and CD3 in yellow. **B** Unsupervised hierarchical clustering showing differences in protein expression profiling for each timepoint for patient 1. **C** Longitudinal plots showing log2 normalized protein expression for B7-H3, Bcl-2, CTLA4, LAG2, Tim3, PD1, PD-L1 and STING. The red boxplots with circles are PanCK− and blue boxplots with triangles are PanCK+ segment

*Patient 1*—Six years after her initial diagnosis of HER2+ left breast cancer and after developing a chest wall recurrence for which she was receiving gemcitabine and trastuzumab, the patient was found to have a right breast mass. A biopsy revealed HER2+ invasive ductal carcinoma and this was the initial biopsy analyzed in our study (Fig. 3A, subpanels a–c). She then received nab-paclitaxel and trastuzumab and underwent a mastectomy that revealed residual disease (second biopsy; Table 1, Fig. 3A, subpanels d–f). Two years later, she developed metastatic disease to the lung (third sample; Table 1, Fig. 3A, subpanels g–i). Expression of immune related proteins clustered by timepoint with the lung metastases having lower overall expression compared to both the primary and recurrent primary samples (Fig. 3A, B).

Immune marker expression was highest in the stromal compartment of the residual disease timepoint suggesting that treatment may have increased immune activity in the TME. We also observed increased expression of B7-H3, Bcl-2, CTLA4, LAG3, Tim-3, PD1, PDL1 and STING in the second sample compared with the first sample (and some differences appear contingent upon the tumor or stroma compartment; Fig. 3C).

**Fig. 4:**
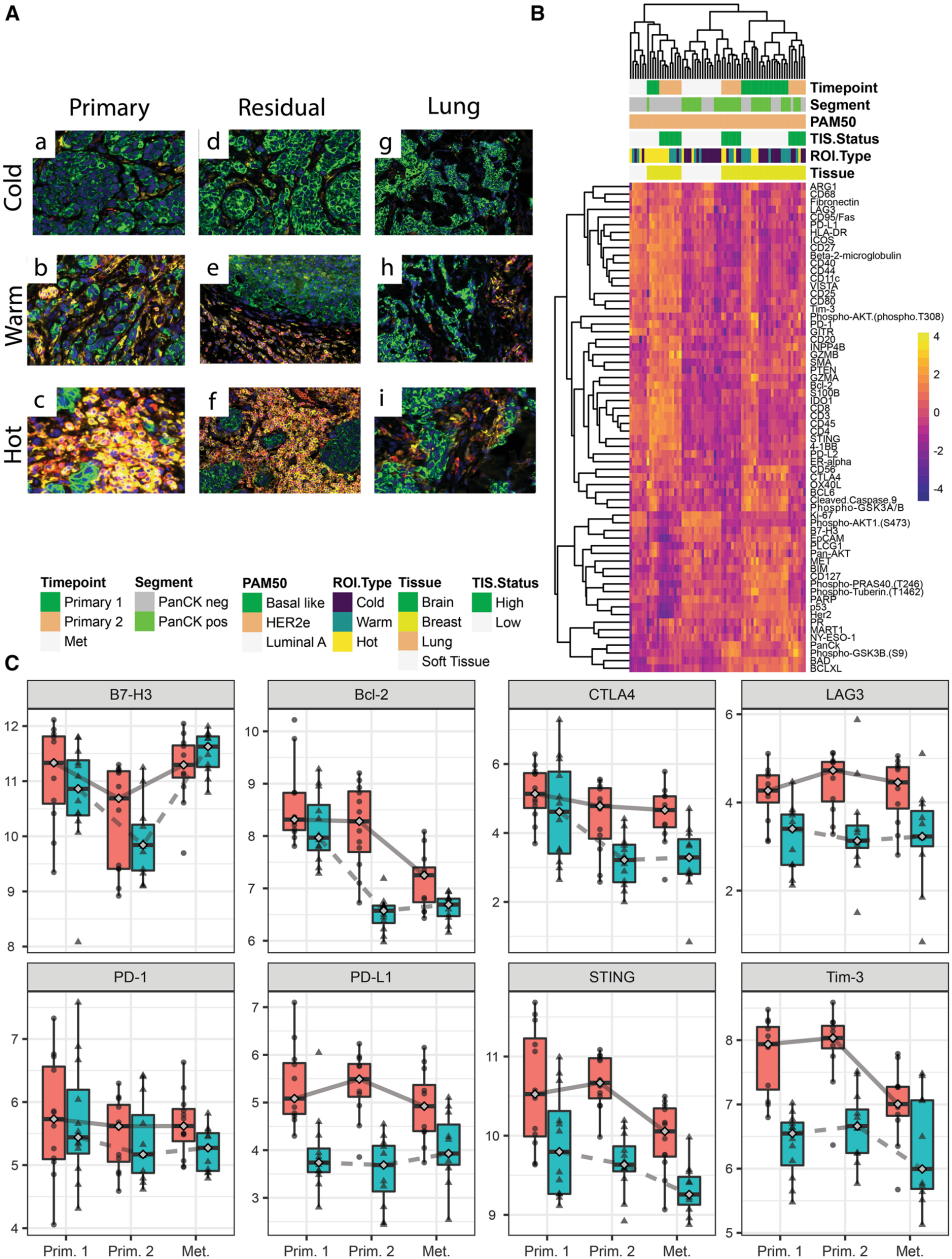
Analysis of primary, residual breast tumor and metastatic samples of patient 2. **A** Shows immune cold, warm and hot regions of interest for the three timepoints for patient 2 at primary, residual breast tumor and metastatic stages. Pan-cytokeratin is stained in green, CD45 in red and CD3 in yellow. **B** Unsupervised hierarchical clustering showing differences in protein expression profiling for each timepoint for patient 2. **C** Longitudinal plots showing log2 normalized protein expression for B7-H3, Bcl-2, CTLA4, LAG2, Tim3, PD1, PD-L1 and STING. The red boxplots with circles are PanCK− and blue boxplots with triangles are PanCK+ segment

*Patient 2—*the first biopsy was collected at diagnosis with early stage HER2+ breast cancer (Fig. 4A, subpanels a–c). She then received neoadjuvant therapy with ado-trastuzumab emtansine and pertuzumab followed by doxorubicin and cyclophosphamide prior to bilateral mastectomies when residual disease was detected and the second sample was collected (Fig. 4A, subpanels d–f). She then received adjuvant radiation as well as trastuzumab and pertuzumab but two years later was found to have a soft tissue chest wall recurrence (third sample; Table 1, Fig. 4A subpanels g–i). Interestingly, for patient 2 the primary time point we observed generally higher overall immune protein expression compared to both the residual and metastatic samples, suggesting reduction in presence of immune cells after neoadjuvant chemotherapy (Fig. 4B). This was particularly striking when looking at specific checkpoint markers in her specific case. Indeed, there was a decrease in the expression of B7-H3, Bcl-2, CTLA4, LAG3, PD1, PDL1 in the second sample (residual disease) and then these increased at recurrence, close to the levels noted at the time of the diagnosis (Fig. 4C).

### Brain metastases

**Fig. 5:**
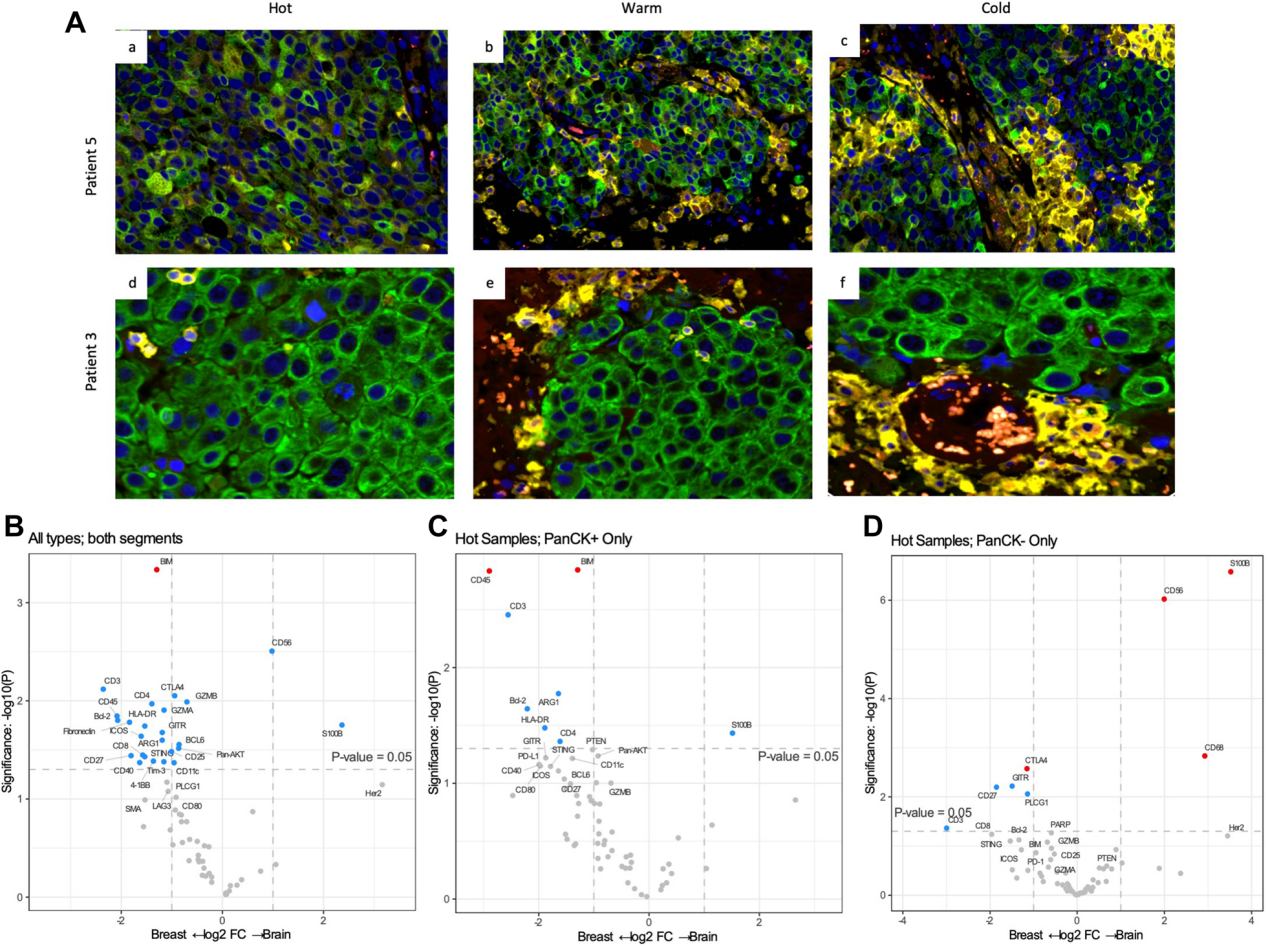
T cell exclusion in brain metastases. **A** Figure shows cold, warm and hot regions of interest of two patients’ brain metastases. PanCK in green, CD45 in red and CD3 in yellow. **B** Differential expression of proteins from all ROI and AOI types primary breast and brain metastases. **C** Differential expression of proteins from all ROI types from the PanCK positive tumor segments comparing primary breast and brain metastases. **D** Differential expression of proteins from all ROI types from the PanCK negative stromal segments comparing primary breast and brain metastases. For **B**-**D** vertical dotted lines represent a onefold log change and the horizontal line marks an unadjusted p-value of p < 0.05. Dots in grey are not significant, dots in blue have an unadjusted p-value of < 0.05 and dots in red have an adjusted p-value of < 0.05

After observing the significant differences in immune-related gene expression from primary disease and brain metastases (Fig. 1C, D), we examined total and tumor/stroma compartment protein expression using DSP (Fig. 5A). Similar to the gene expression results, we found that total immune infiltration (CD8, CD4, CD45), cytotoxicity (GRZMA, GZMB), T cell activation (CTLA4, GITR, TIM3, CD25, LAG3, CD27, OX40L, PD-L1, PD1, PD-L2) and antigen presenting cell activation presence (CD11c, HLA-DR, CD80, CD40) were significantly lower in brain metastases compared to primary tumors in all compartments and ROI types (Fig. 5A, B). Interestingly we found in the immune hot ROIs that protein expression and immune cell presence was drastically different between the tumor and stroma compartments for primary and brain metastases. The tumor compartment of primary samples had significantly higher expression of immune cell markers CD45, CD8, ARG1, GZMB, BIM, BCL2, showing brain metastases have very few immune cells infiltrating in the tumor epithelium (Fig. 5C). In contrast, when we examined the stromal compartment of immune hot ROIs, expression of overall immune markers (CD3, CD8) did not reach significance, suggesting there are immune cells in the stroma of primary and brain metastases are similar but that they are not able to infiltrate the brain metastasis tumor epithelium (Fig. 5D). The stromal compartment of primary tumors did have higher expression of lymphocyte activation markers (CD27, GITR, CD25, cytotoxicity (GRMA, GRMB), B cell markers (BIM, BCL2) and checkpoint molecules (PDL2, PDL1, CTLA4) (Additional file 1: Table SVI).

## Discussion

In this study we described the TME and gene expression profiling of 15 HER2+ breast tumors. We observed that primary samples had higher immune cells relative to metastatic sites. Primary breast tumor samples also had higher expression of immune activation and checkpoint markers when compared with metastatic samples. These findings suggest that metastatic tumors are less immunogenic than primary breast cancers and could explain the limited efficacy of ICIs in metastatic HER2+ breast cancer [18, 19, 32]. Similar findings have been described in TNBC in which primary tumors tend to have higher numbers of immune cells [12, 14].

Metastatic tumors had lower expression of immunotherapy response predictive signatures, relative to primary tumors. Figure 1E shows the immune protein expression of the 15 samples and the metastatic sites had lower expression of these markers, suggesting that the primary tumors are more immunogenic than the metastatic ones. When looking at gene expression profiling in this cohort, primary tumors had greater B cell abundance. However, the protein profiling provides additional spatial context. Namely, B cells were increased in stroma and not in tumor compartments. This suggests a possible role of tertiary lymphoid tissue formation and not B-cell TILs. The role of tertiary lymphoid tissue in the prognosis of solid tumors remains controversial [33]. These findings underscore the importance of DSP in understanding not only the cell signatures present in the tumor but also their location as their function and prognostic significance may variate in different settings.

Our cohort was unique in that we had paired samples of five patients. When we analyzed the samples of the three patients with two timepoints and their treatment history, patients 4 and 7 had a somewhat similar clinical presentation. Patient 8 had a HER2+ and an early TNBC and years later developed a triple negative local recurrence. When analyzing protein expression of immune markers, we found similar patterns for patients 4 and 7 with an increase in expression Bcl-2, CD20, LAG3, MET (PanCK-), PTEN, AKT (PanCK-) after therapy (Additional file 1: Figure S3). We found that metastatic samples had lower numbers of immune cells and immune activation markers than primary tumors. It is possible that patients 4 and 7 had an increase in activation markers as an effect of therapy. The protein expression analysis of patient 8 is different, however this patient was heavily pretreated and the samples are over 15 years apart and different subtypes. The HER2+ sample had higher expression of several immune markers relative to the TNBC sample, including CD20, CTLA4, MET, Tim3 and AKT.

Patients 1 and 2 had three timepoints. Figures 3A and 4A illustrate the higher levels of immune cells noted in the primary tumor when compared to metastatic sites in patients 1 and 2. Although the patients were receiving different therapies these findings were consistent. Similar findings have been reported in TNBC, primary triple negative tumors have shown to have high numbers of immune cells, such as CD8 and naïve CD4 [12]. The protein expression analysis for patient 1 revealed higher expression of potentially targetable immune markers after the initial therapy (Timepoint 2), similar to what we saw with patients 4 and 7. However the markers were lower in the metastatic sample (Timepoint 3), suggesting that decreased expression of immune markers allows for immune escape of the cancer cells into distant organs, such as the lungs. This pattern was not seen in patient 2 who had soft tissue chest wall metastases. Figures 3C and 4C illustrate the variable expression of potentially targetable immune related markers in the different timepoints for patients 1 and 2.

DSP allows for a comprehensive analysis of protein expression by which we were able to assess immune markers that have been proposed as markers of response to ICI, such as PD-L1, and other potential therapeutic markers, such as B7H3 or LAG3. Larger studies are needed to determine if this technology can be used to guide treatment decisions in the future with the goal to provide individualized care to our patients.

The breast cancer intrinsic subtypes have shown to provide prognostic [34] and predictive [35] information in breast cancer. There is growing interest to understand the role of these subtypes in clinical practice. In this study we analyzed PAM50 for breast cancer subtypes, 40% of the patients had HER2-E tumors, 33% Luminal A and 27% Basal like. HER2-E tumors had higher expression of the ERBB2 and proliferation subtypes, while Luminal A tumors had higher expression of immune activation and checkpoint markers. Suggesting that these tumors may benefit from ICI but further confirmatory investigation is needed.

ICIs have shown central nervous system penetration in lung cancer [34, 35] and melanoma [36, 37] however, patients with unstable and untreated breast cancer brain metastases have not been included in ICI breast cancer studies [15, 16]. Our cohort included two HER2+ brain metastases samples. These samples had the lowest numbers of immune cells and a low TIS. It is difficult to determine if the use of systemic therapy had an impact in the TME and gene expression profiling of these samples. Further studies are needed to determine the optimal combination of treatment for HER2+ breast cancer brain metastases and if there is a role for ICIs in this setting.

Several studies have shown efficacy of the combination of ICI with HER2-targeted agents in PD-L1 positive HER2+ breast cancer [18, 19, 40]. Multiple trials are ongoing to determine the role of PD-1 and PD-L1 blockers in HER2+ breast cancer. There is also growing interest in studying other potential targets in breast cancer. Examples of immune targets under investigation for the treatment of specific breast cancer subtypes include B7-H3, LAG3 and CTLA4; there is also interest in assessing the role of Bcl-2 inhibition in selected patients with breast cancer. We describe variations in expression of these proteins in paired samples suggesting that there may be a role for protein expression testing at different stages of the disease to guide therapy which could allow us to provide personalized care for our patients [41].

Limitations of this study include the small sample size, which restricts the correlation between the tumor immune characteristics, treatment, and outcomes. Even though our study is small the strength is the paired samples. Also, we will contribute our data to the GeoMx Breast Cancer Consortium for a meta-analysis with other investigators examining primary and metastatic breast cancer to increase the data set. Another limitation is that the ROI selection might not capture the heterogeneity of the entire tissue. We chose to look at DSP with proteins, other analyses can be done using in situ RNA examination of up to 18,000 genes using DSP, which gives spatially resolved information on signaling pathways as opposed to the bulk gene expression presented in this paper.

This study underscores the importance of spatial resolution of the tumor or stroma within the TME to see distinct differences in immune activation markers. A better understanding of the TME and gene expression profiling of HER2+ breast cancer will allow us to tailor the treatment of patients to continue to improve outcomes.

## Supplementary Information

**Additional file 1.** Additional figures and tables.

## Data Availability

The datasets generated and/or analyzed during the current study are not publicly available but are available from the corresponding author on reasonable request. Data partially presented in the 2021 AACR Virtual Annual Meeting (April 9–14, 2021).

## Abbreviations

TME: Tumor microenvironment
HER2: Human epidermal growth factor receptor 2
PBT: Primary breast tumors
BM: Brain metastases
TIS: Tumor inflammation signature
TNBC: Triple negative breast cancer
ICI: Immune checkpoint inhibitors
PD-L1: Program cell death ligand 1
DSP: Digital spatial profiling
FFPE: Formalin fixed paraffin embedded
IRB: Institutional Review Board
ER: Estrogen receptor
PR: Progesterone receptor
H&E: Hematoxylin and eosin
HK: Housekeeping
PanCK: Pan-Cytokeratin
ROIs: Regions of interest
AOIs: Areas of illumination
BH: Benjamin-Hochberg
