# Supplementary material for: The tumor immune microenvironment of primary and metastatic HER2− positive breast cancers utilizing gene expression and spatial proteomic profiling
Source: J Transl Med. 2021 Nov 27;19:480. doi: 10.1186/s12967-021-03113-9 (PMC8626906; doi:10.1186/s12967-021-03113-9)

Additional file 1: Table S1: 70 protein targets including housekeeping proteins and isotype controls used for GeoMx DSP


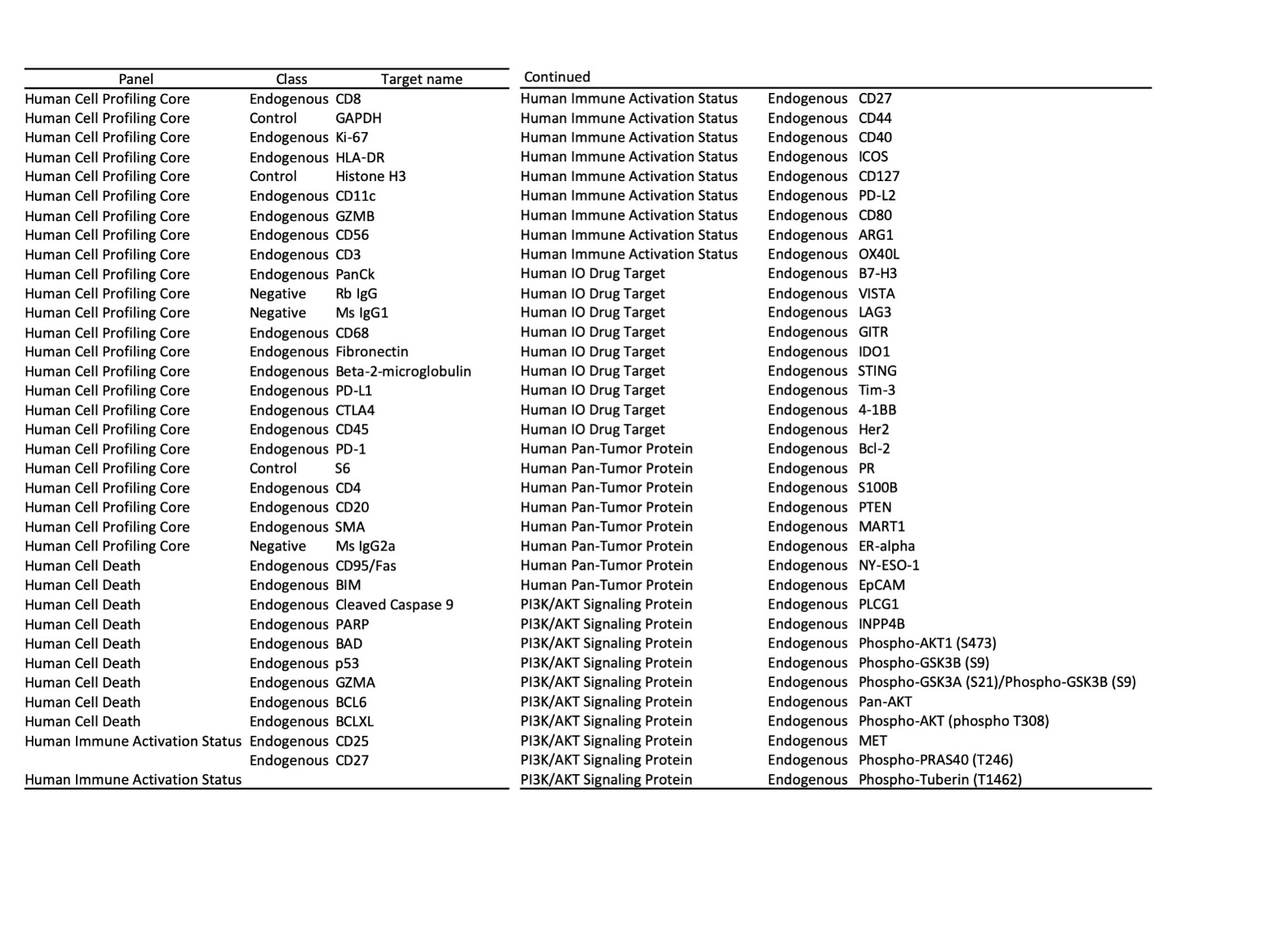


Additional file 1: Table S2: Differential expression of immune signatures in primary and metastatic tumors


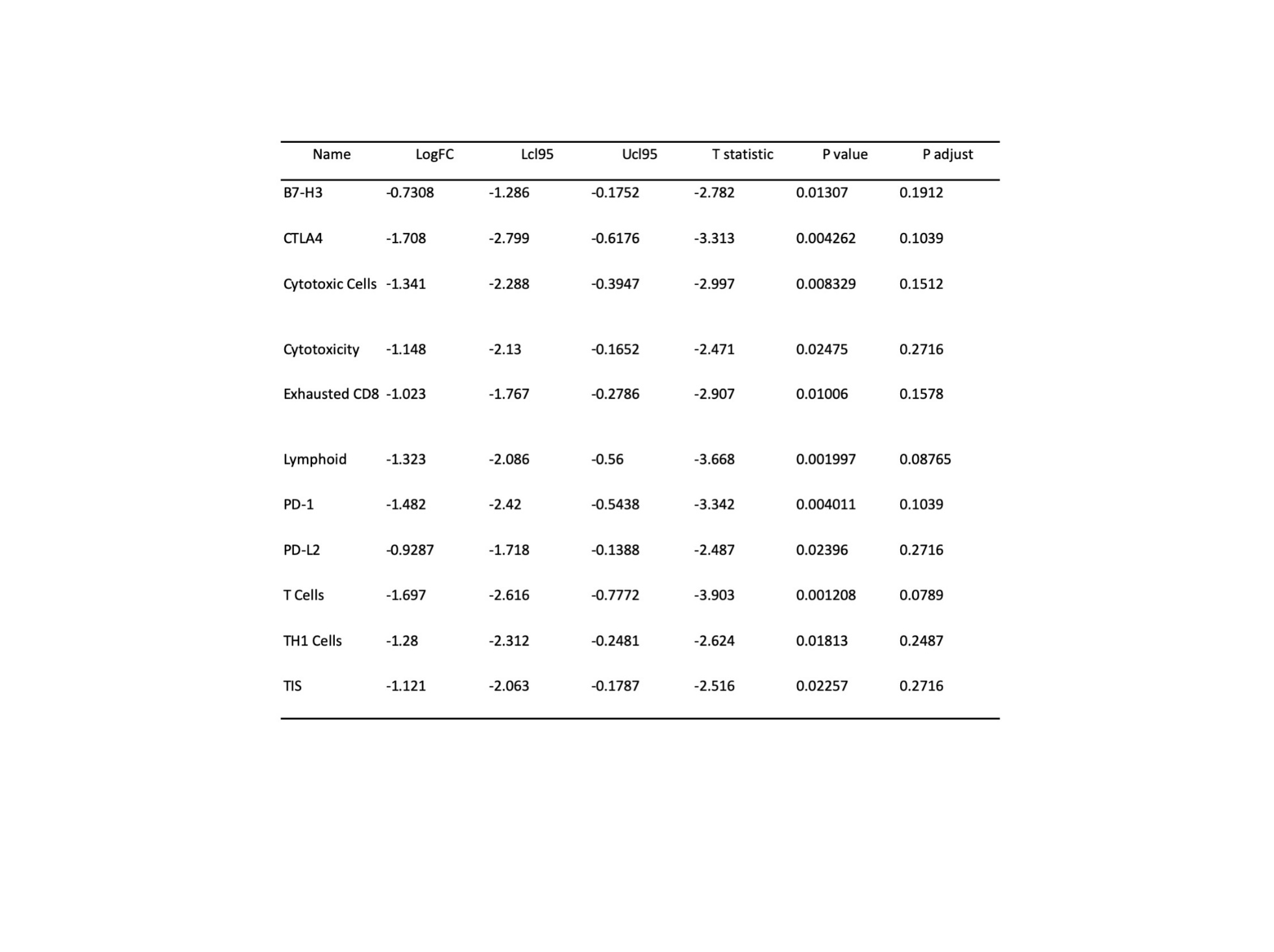


Additional file 1: Table S3: Differential expression of individual genes in primary and metastatic tumors


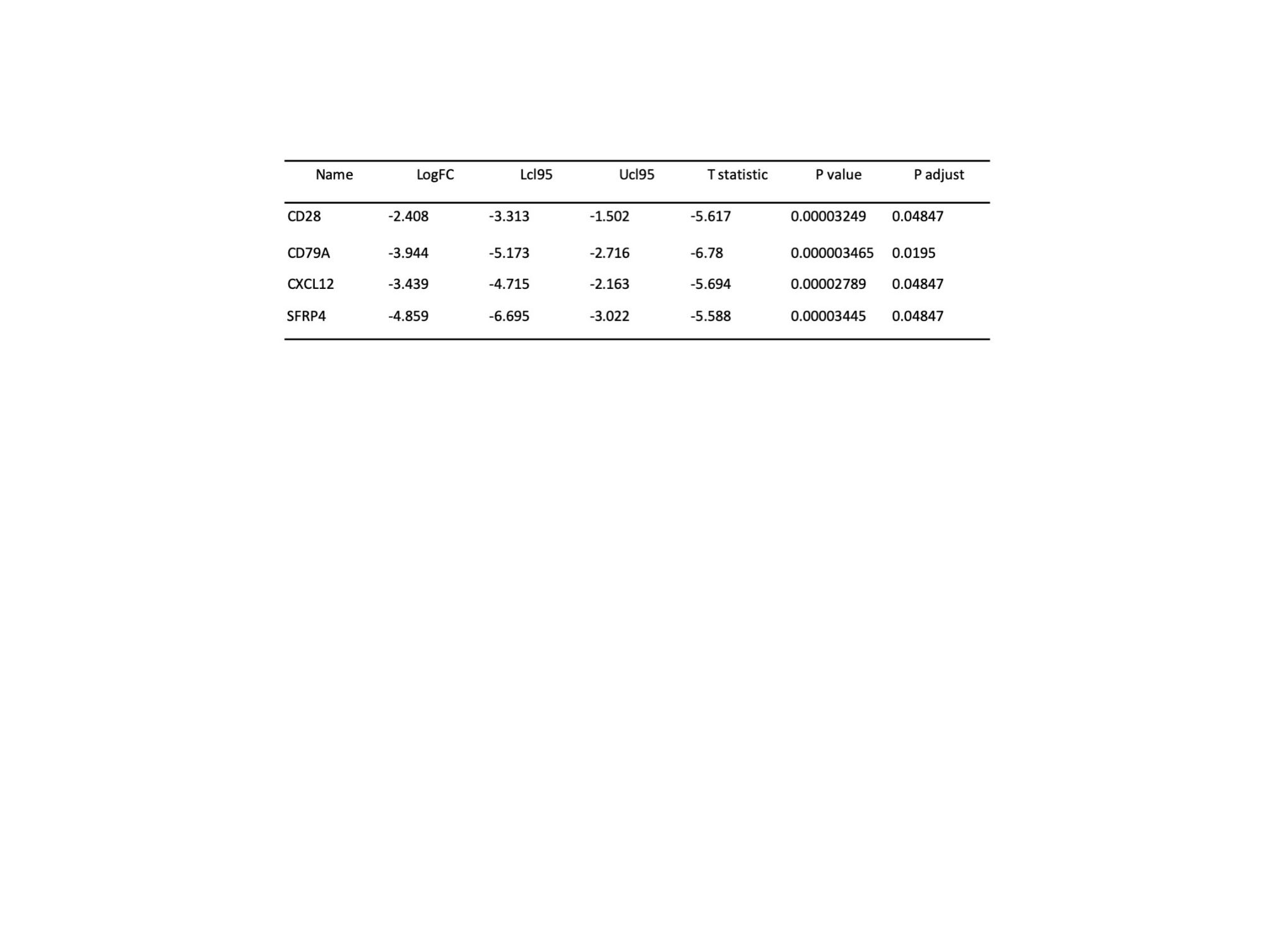


Additional file 1: Table S4: Differential expression of immune signatures in primary and brain metastases


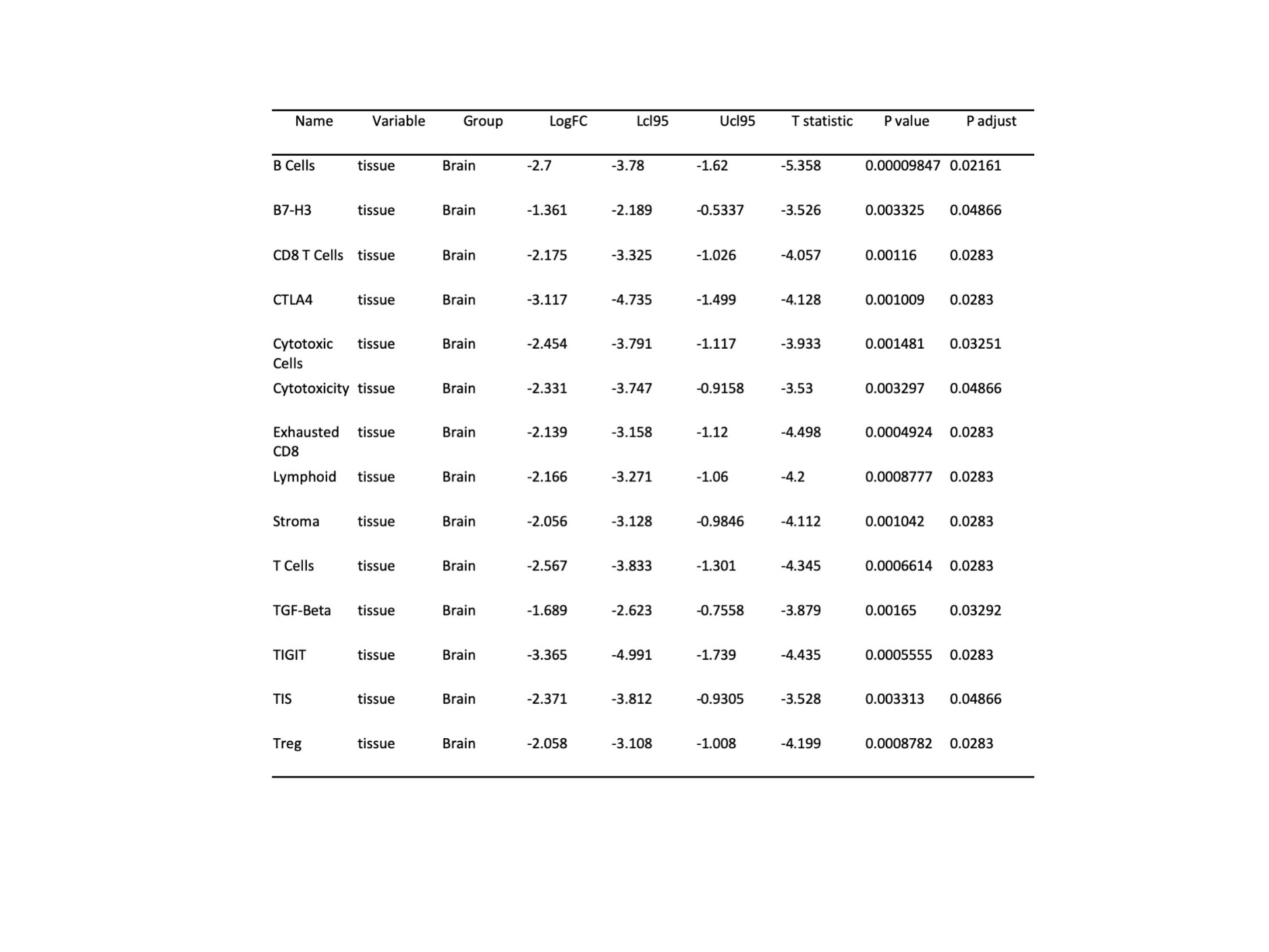


Additional file 1: Table S5: Differential expression of immune markers in different regions of interest (comparing primary and metastatic samples)


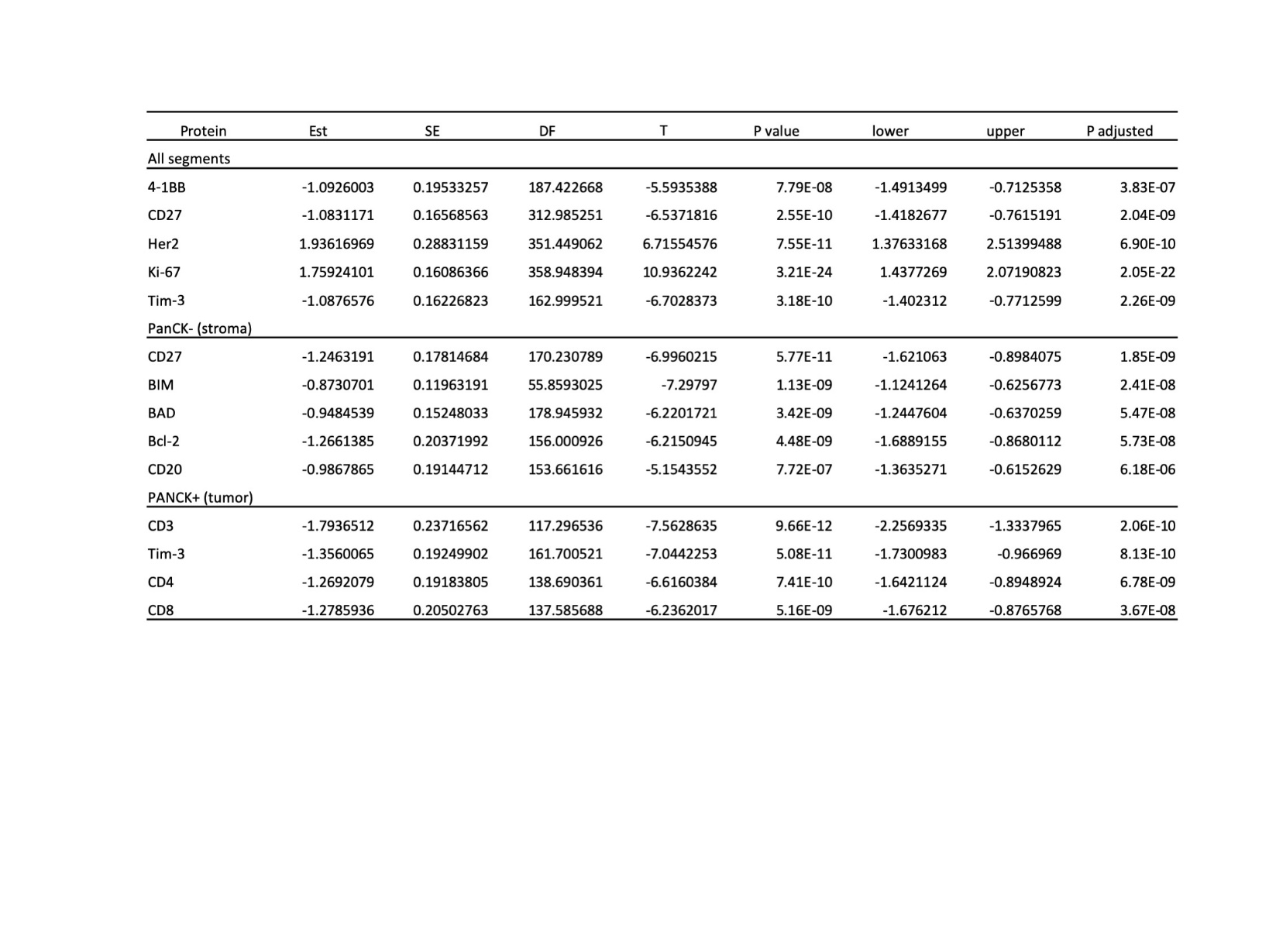


Additional file 1: Table S6: Differential expression of immune markers in different regions of interest (comparing primary tumors and brain metastases)


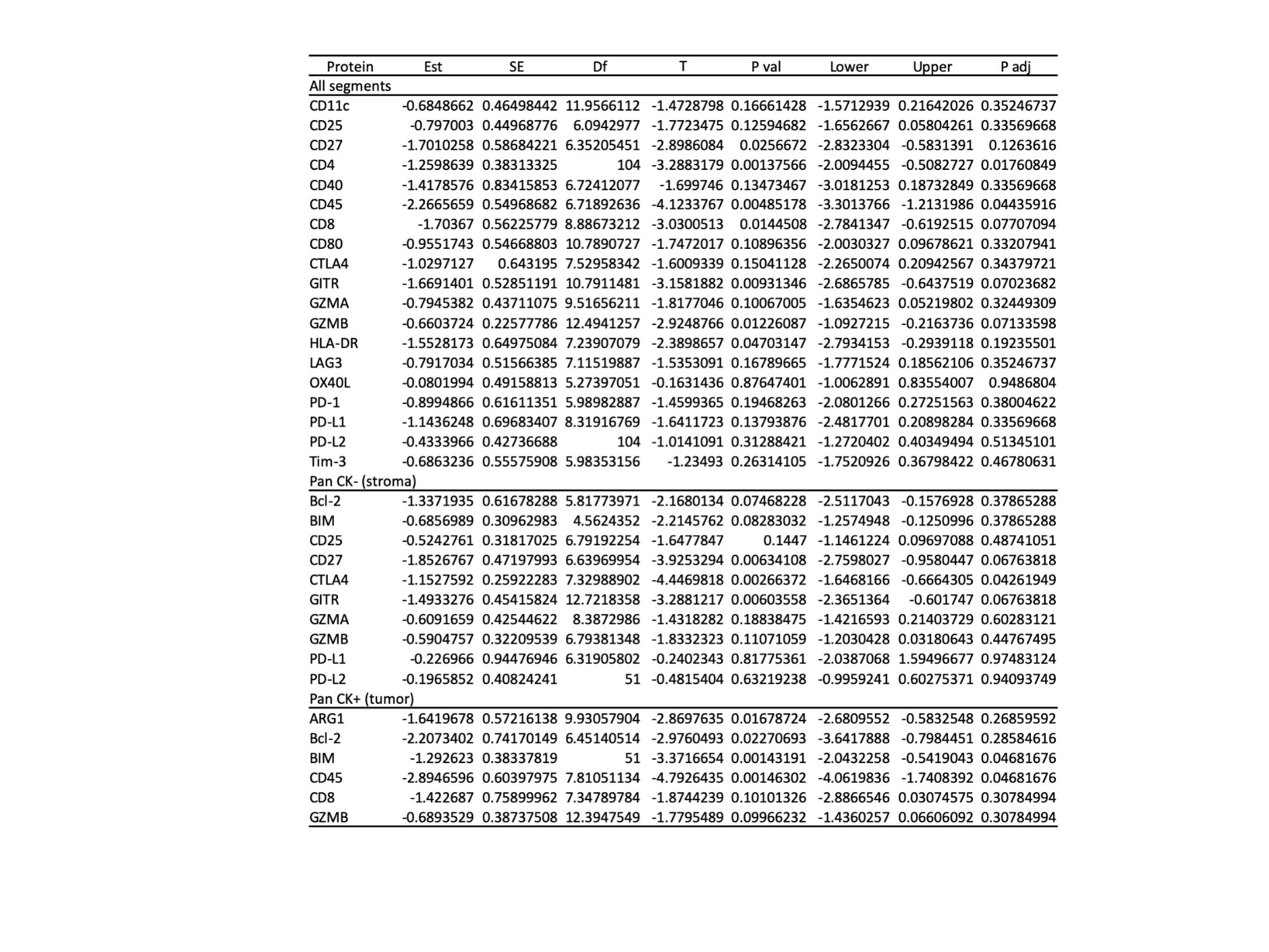


Additional file 1: Figure S1: The signal-to-noise ratio for each of the targets included in GeoMx DSP


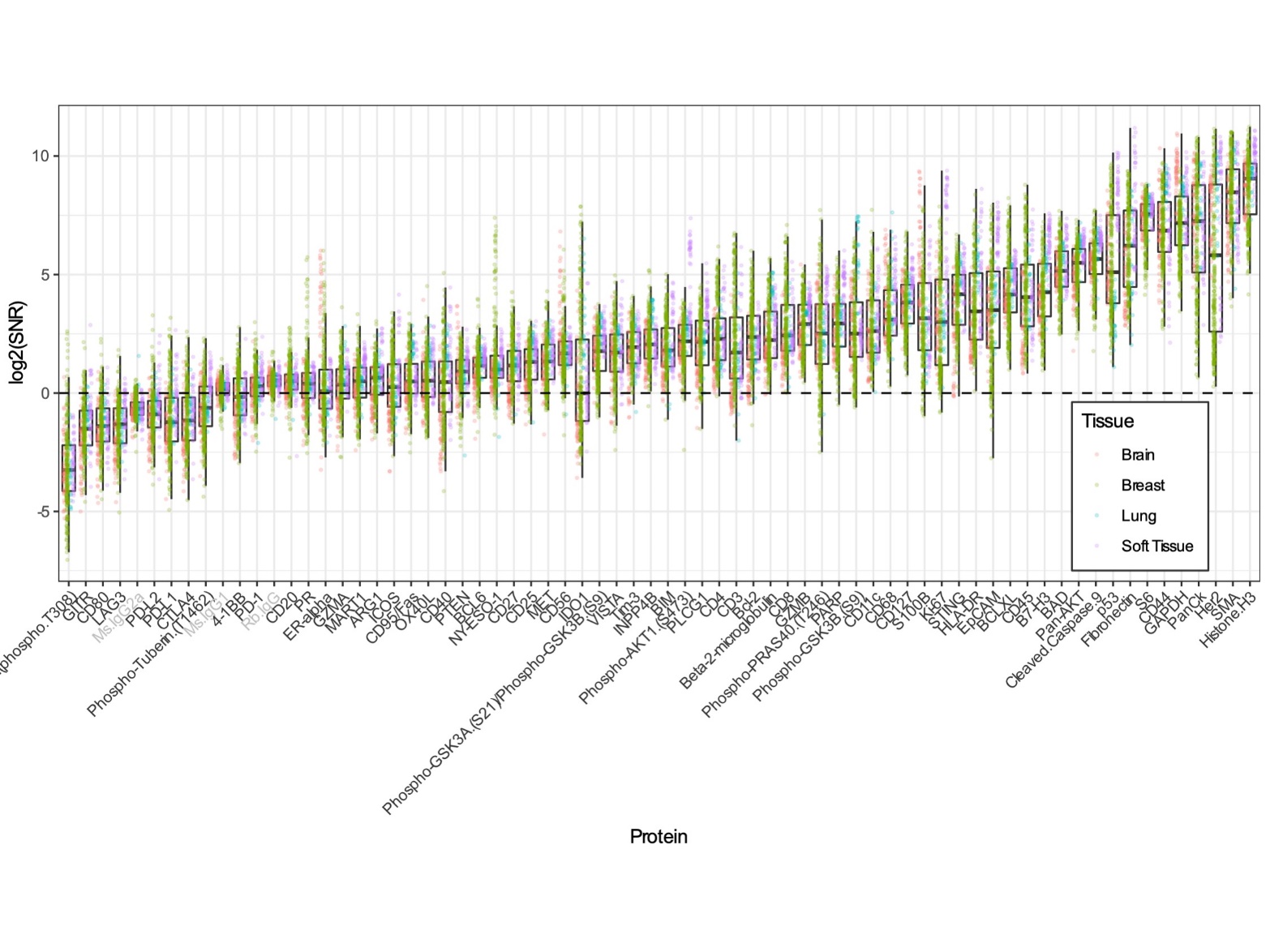


Additional file 1: Figure S2: Treatment and disease history for patients 1 and 2


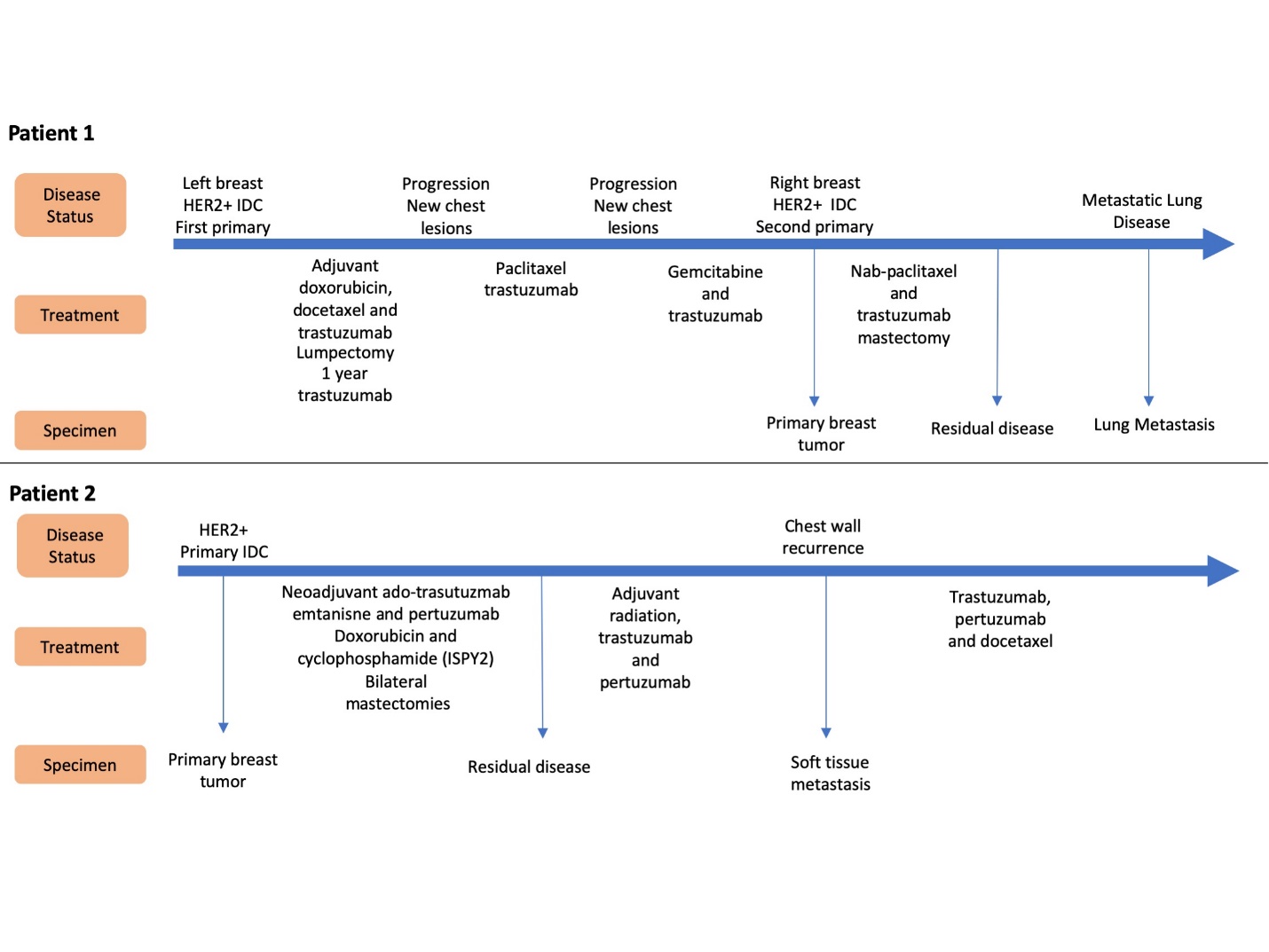


Additional file 1: Figure S3: Treatment and disease history for patients 4, 7 and 8 and longitudinal plots showing protein expression of immune markers. Pink represents expression in PanCK negative stroma and teal is PanCK positive tumor AOIs


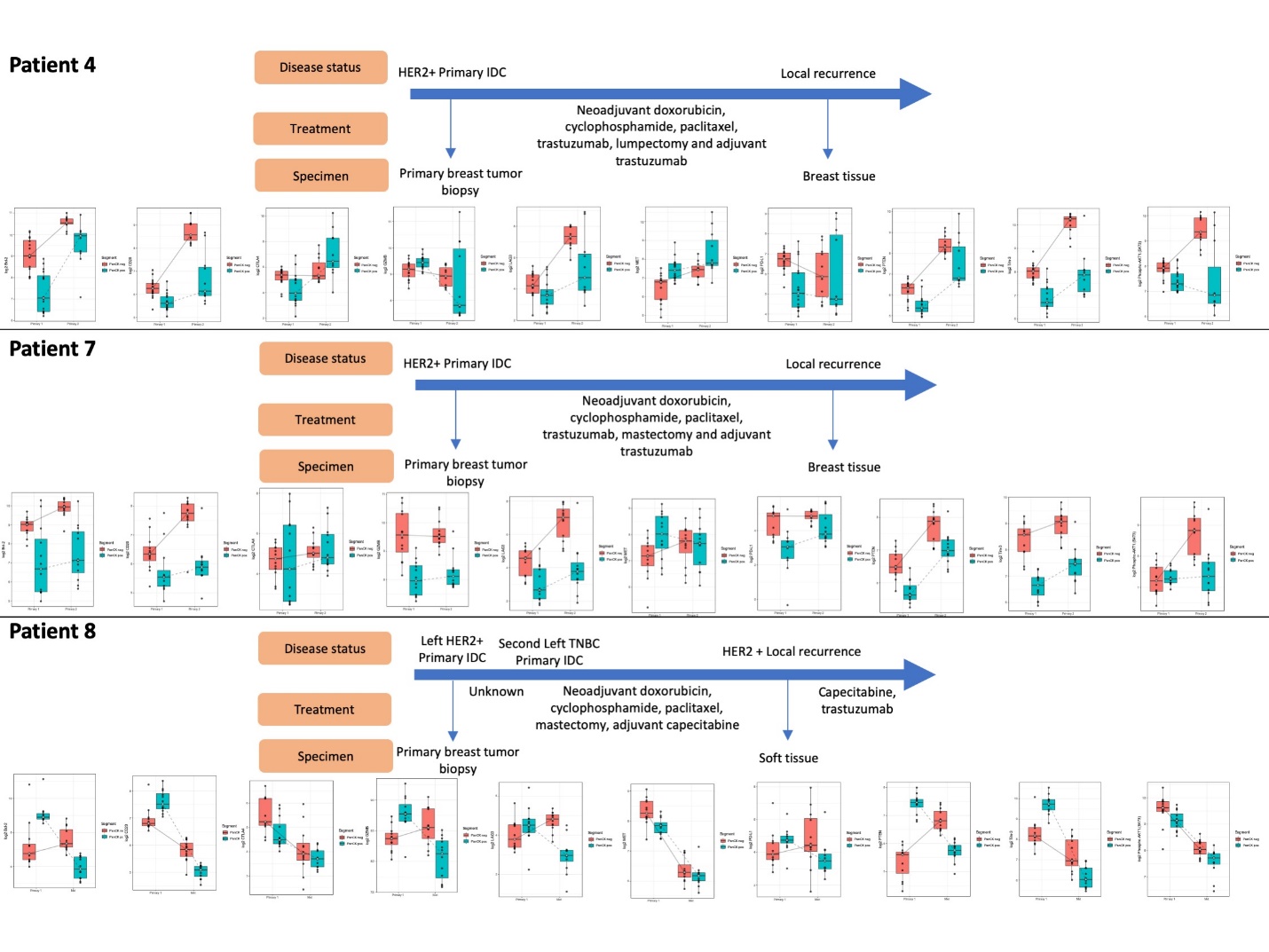


Additional file 1: Figure S4: Gene signatures for each of the PAM50 subtypes


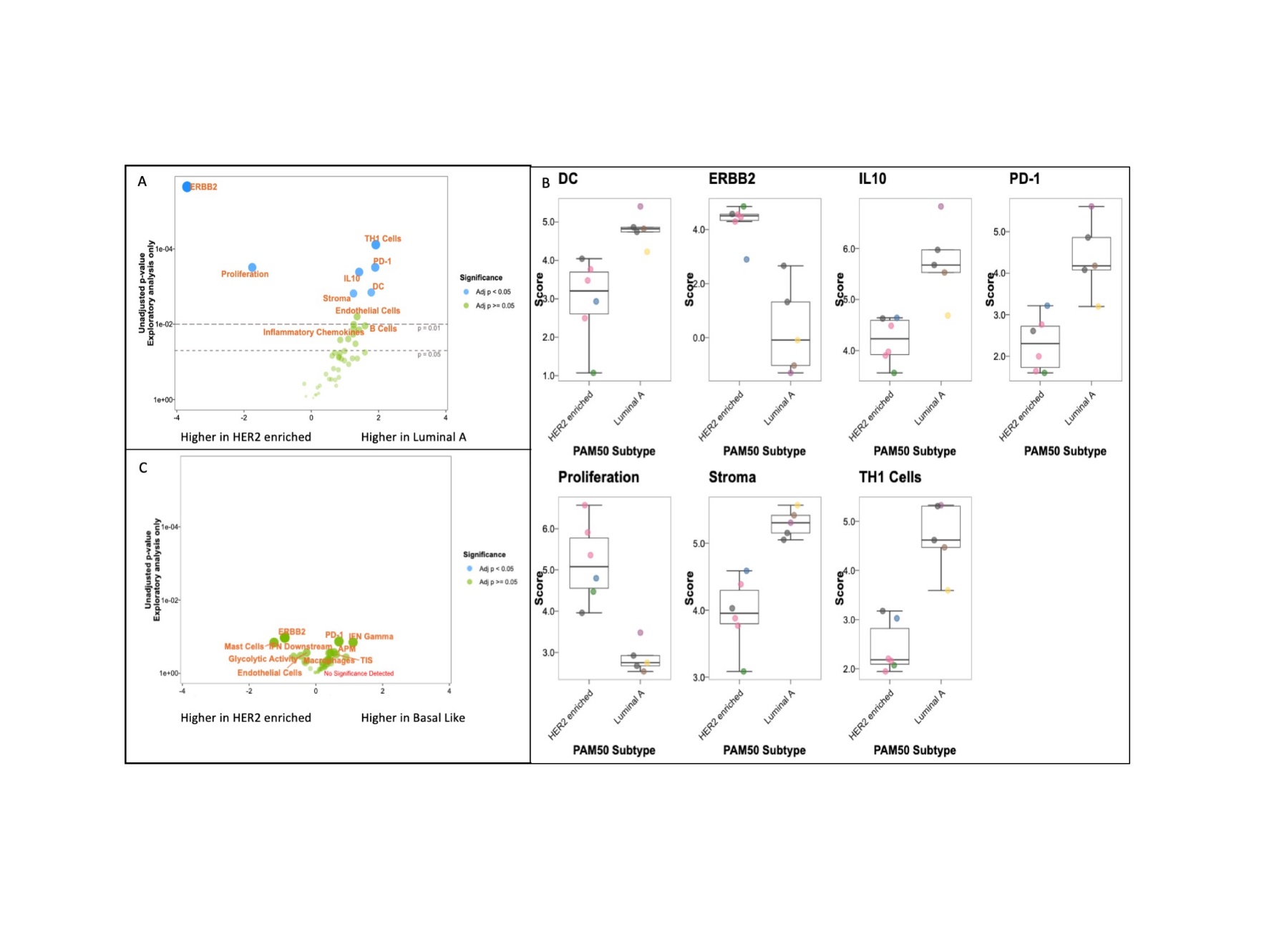

Supplement: Supplementary file 1 — Additional file 1. Additional figures and tables. [file 12967_2021_3113_MOESM1_ESM.docx]
